# Successful application of whole-liver irradiation in a patient with small cell lung cancer with diffuse liver metastasis: a case report

**DOI:** 10.1186/s13256-026-06340-0

**Published:** 2026-07-08

**Authors:** Takahiro Ogasawara, Yukihiro Toi, Yusuke Yamazaki, Tsuyoshi Doman, Soichiro Minami, Akane Narumi, Shin Saito, Tetsuo Odaka, Kana Ono, Hisashi Shimizu, Jun Sugisaka, Tomoiki Aiba, Sachiko Kawana, Shinsuke Yamanda, Yuichiro Kimura, Misako Miwa, Shunichi Sugawara

**Affiliations:** 1 Department of Pulmonary Medicine, Sendai Kousei Hospital, 1-20 Tsutsumidori-Amamiyamachi, Aoba-Ku, Sendai, Miyagi 981-0914 Japan; 2 Department of Radiology, Sendai Kousei Hospital, Miyagi, Japan

**Keywords:** Small cell lung cancer, Liver metastases, Whole-liver irradiation, Palliative radiotherapy, Hepatic dysfunction, Chemotherapy

## Abstract

**Background:**

Small cell lung cancer (SCLC) is an aggressive malignancy with a strong propensity for metastasis, often leading to liver metastases associated with severe hepatic dysfunction. Whole-liver irradiation (WLI) is a palliative treatment option for patients with diffuse liver metastases, especially in cases, where systemic chemotherapy is contraindicated.

**Case presentation:**

We report the case of a 74-year-old Japanese man of East Asian ethnicity with extensive-disease small cell lung cancer (ED-SCLC) and diffuse liver metastases associated with severe hepatic dysfunction following first-line chemotherapy. The patient, initially deemed unsuitable for chemotherapy due to hepatic dysfunction, complained of severe abdominal distension and underwent WLI at a dose of 8 Gy in a single fraction for symptom palliation. Within 2 weeks, the patient showed significant improvement in hepatic function and symptoms. This improvement coincided with sufficient hepatic recovery to permit the subsequent administration of second-line chemotherapy. WLI may provide symptomatic relief for patients with liver metastases and may be associated with hepatic recovery sufficient to allow further systemic treatment in selected cases. Although its role is limited by liver radiation tolerance, advancements in radiotherapy techniques, such as stereotactic body radiation therapy, offer promising complementary approaches. This case highlights the potential role of WLI as a palliative option that may be associated with hepatic recovery sufficient to permit subsequent systemic therapy in selected patients with severe hepatic dysfunction caused by liver metastases.

**Conclusions:**

WLI may serve as a palliative option for patients with ED-SCLC and hepatic dysfunction and, in selected cases, may be associated with hepatic recovery sufficient to permit subsequent systemic treatment. Further research is warranted to establish optimal protocols and patient selection criteria.

## Background

Small cell lung cancer (SCLC) is a highly aggressive malignancy characterized by rapid progression and a strong propensity for metastasis. Liver metastases frequently occur in advanced stages of SCLC and are associated with severe hepatic dysfunction, reduced quality of life, and poor prognosis. Although the administration of systemic chemotherapy to patients with severe hepatic dysfunction is sometimes challenging, some cases present a dramatic response to this treatment strategy [1]. For selected cases, hepatic arterial infusion chemotherapy has also shown a beneficial effect in controlling liver metastases, improving quality of life, and potentially extending survival [2, 3]. Early diagnosis and prompt initiation of chemotherapy are crucial for improving patient outcomes [1].

Despite these treatment advances, the prognosis of patients with SCLC with liver metastases remains poor. A retrospective study reported a median overall survival of 10.97 months for patients receiving first-line therapy, with adverse prognostic factors, such as Eastern Cooperative Oncology Group performance status ≥ 2 and mixed SCLC histology, associated with worse outcomes [4, 5]. Integrating systemic chemotherapy with local therapies, including radiotherapy, has been proposed as a potential strategy to improve outcomes in this patient population [4].

Whole-liver irradiation (WLI) has emerged as a potential treatment option for liver metastases in SCLC, particularly in cases complicated by chemotherapy resistance or severe hepatic dysfunction. Edyta et al. [6] reported that palliative WLI (mean dose 17 Gy in 5–12 fractions) effectively alleviated symptoms such as abdominal pain and improved the quality of life of patients with liver metastases. Similarly, Ito et al. [7] demonstrated that WLI at a dose of 8 Gy in a single fraction improved liver enzyme levels and symptoms in patients with diffuse liver metastases, especially those without prior local treatments, such as radiofrequency ablation or transcatheter arterial embolization. Furthermore, low-dose WLI has been shown to achieve tumor shrinkage and symptomatic relief in patients with treatment-resistant SCLC and multiple liver metastases [8].

Recently, several reports have reaffirmed the clinical relevance of WLI. Ogoshi et al. [9] reported a case of diffuse liver metastases from castration-resistant prostate cancer that responded dramatically to single-fraction 8 Gy whole-liver radiotherapy, achieving rapid biochemical improvement. Furthermore, the CCTG HE1 phase III trial demonstrated that radiotherapy to the whole or near-whole liver provided significant pain relief compared with that of the best supportive care [10]. These contemporary studies and our findings suggest that WLI remains a feasible palliative strategy for selected patients with severe hepatic dysfunction and may, in some cases, be associated with hepatic recovery sufficient to permit subsequent systemic therapy when chemotherapy is initially contraindicated.

Herein, we report a case of advanced SCLC with diffuse liver metastases and severe hepatic dysfunction following first-line chemotherapy. The patient complained of severe abdominal distension and underwent WLI as a palliative intervention, resulting in significant improvement in hepatic function and symptoms. This improvement coincided with sufficient hepatic recovery to permit subsequent systemic chemotherapy. We report this case to illustrate the clinical potential of WLI in similar challenging scenarios.

## Case presentation

### Case

A 74-year-old Japanese man of East Asian ethnicity.

### Medical history

The patient’s medical history included hypertension, chronic pancreatitis, gallstones (status post-cholecystectomy), colorectal polyps, and cataracts. His lifestyle history revealed a 36-year smoking history of 20 cigarettes per day (from age 20 to 56). At presentation, his activities of daily living were independent, and his performance status was 1.

### Diagnostic course

In September 2018, the patient visited a local physician with nocturnal cough as the primary complaint. A chest X-ray revealed an abnormal shadow in the right middle lung field, prompting a referral to our hospital. Subsequent investigations, including imaging and biopsy, confirmed the diagnosis of extensive-disease small cell lung cancer (ED-SCLC), classified as cT4N3M1c (lymph nodes, liver, and bone), Stage IV (8th edition).

### Treatment

**Fig. 1:**
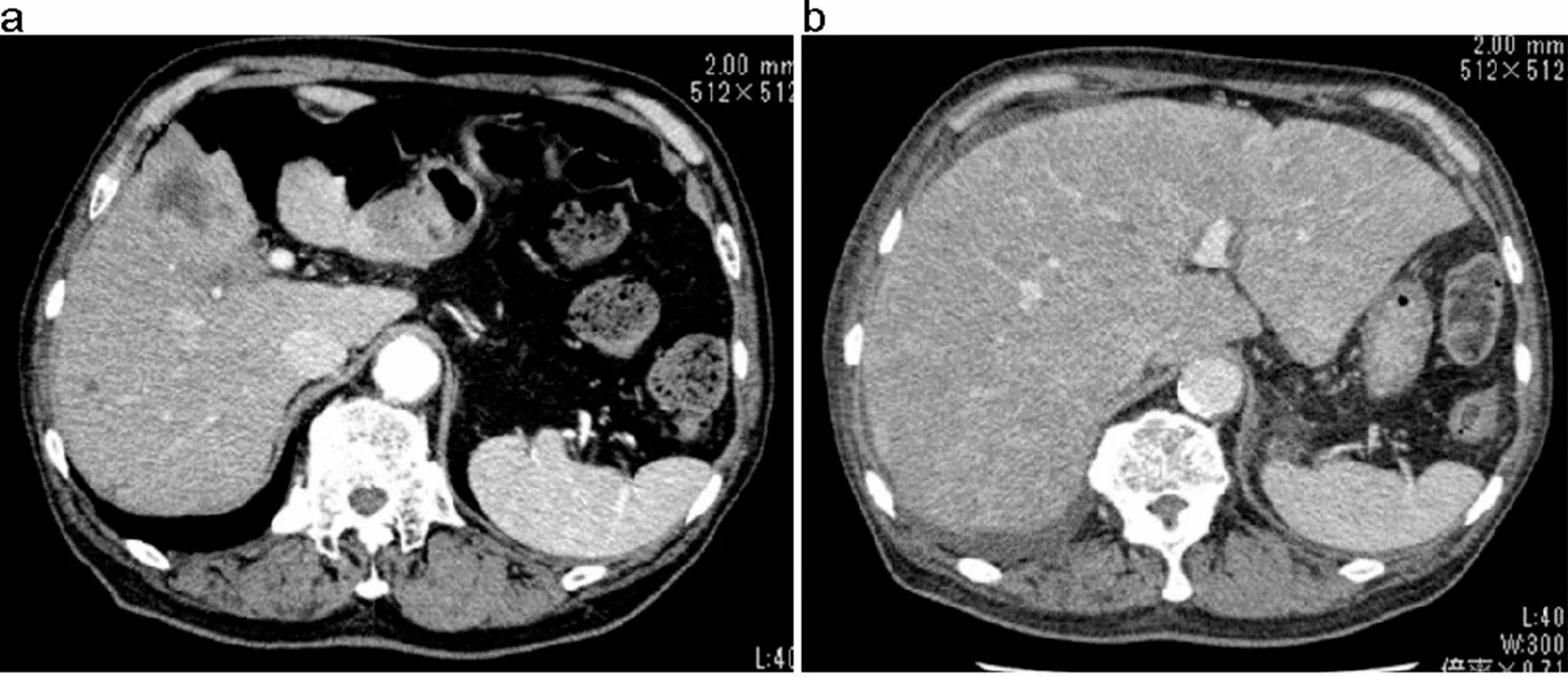
CT scans at disease progression, after chemotherapy, and before whole-liver irradiation. The CT image in **a** shows no significant changes in hepatic metastases, with mild hepatic dysfunction (Grade 1). In contrast, the CT image in panel **b** demonstrates marked hepatomegaly with multiple low-density lesions, consistent with progressive diffuse liver metastases and worsening hepatic dysfunction (Grade 3)

**Fig. 2:**
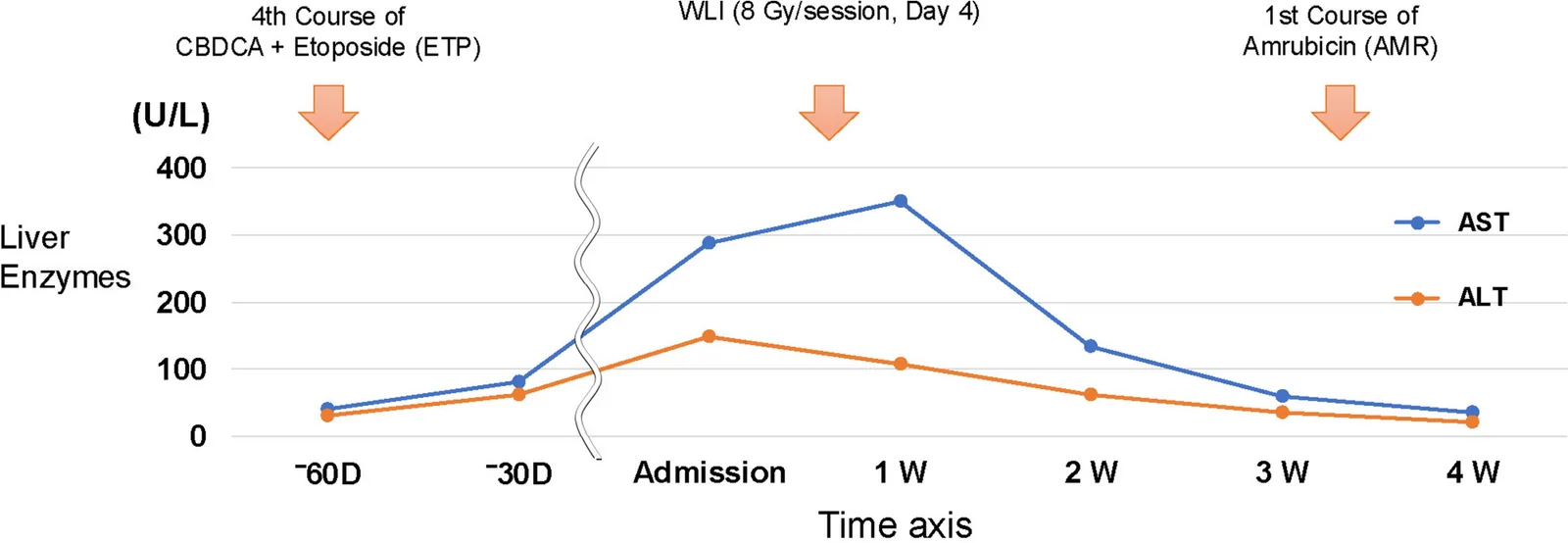
Changes in liver function test results during hospitalization. Laboratory findings on admission showed Grade 3 hepatic dysfunction, with total bilirubin (T-Bil) 4.46 mg/dL, aspartate aminotransferase (AST) 289 U/L, alanine aminotransferase (ALT) 148 U/L, alkaline phosphatase (ALP) 1567 U/L, and γ-glutamyl transpeptidase (γ-GTP) 1695 U/L. Whole-liver irradiation (WLI, 8 Gy) was performed on March 15, 2019, day 4 of hospitalization (arrow). Although liver enzyme levels initially increased after irradiation, they showed marked improvement by the second week. The patient also reported subjective improvement in abdominal distension and appetite, and amrubicin chemotherapy was initiated on April 1, 2019, 17 days after WLI. Units: AST, ALT, ALP, γ-GTP (U/L); T-Bil (mg/dL). The *x*-axis represents days after admission

The patient began first-line chemotherapy with carboplatin at area under the curve 5 intravenously on day 1 and etoposide at 80 mg/m^2^ intravenously on days 1–3, completing four cycles on January 4, 2019. The best response was partial response. After the fourth cycle of first-line chemotherapy, abdominal computed tomography (CT) scans showed no significant changes in hepatic metastases, and hepatic dysfunction was mild, corresponding to Grade 1 according to the Common Terminology Criteria for Adverse Events (CTCAE) version 5.0 [11]. However, on March 11, 2019, approximately 9 weeks after completion of first-line chemotherapy, the patient was readmitted to our hospital with worsening dyspnea and abdominal distension. A CT scan at the time of readmission showed significant hepatomegaly due to multiple liver metastases, and we diagnosed the condition as progressive disease (Fig. 1). Before WLI, laboratory tests showed marked hepatic dysfunction, with total bilirubin of 4.46 mg/dL, AST of 289 U/L, ALT of 148 U/L, and ALP of 1567 U/L, consistent with severe hepatic impairment (Child–Pugh class C). Serologic tests for hepatitis B surface antigen and hepatitis C antibody were negative. There was no history of recent alcohol intake or exposure to hepatotoxic drugs. Therefore, viral, alcoholic, and drug-induced hepatitis were excluded, and the hepatic dysfunction was attributed to diffuse metastatic involvement of the liver. The patient exhibited worsening symptoms, including abdominal distension, anorexia, and elevated liver enzyme levels corresponding to CTCAE Grade 3, which was an obstacle to administering systemic chemotherapy. After consultation with the radiologist, WLI at a dose of 8 Gy in a single fraction was performed for symptom relief on March 15, 2019, the 4th day of admission. The treatment was delivered using three-dimensional conformal radiotherapy. The planning target volume was defined as the whole liver with a 5-mm margin. No predefined formal dose constraints were applied to organs at risk, including the kidneys, stomach, and bowel. Patient positioning was verified using linac radiography before irradiation. Although liver enzyme levels initially increased over the following days, they showed marked improvement by the second week of admission (Fig. 2). The patient also reported subjective improvement in abdominal distension and appetite within 2 weeks after WLI. With the normalization of hepatic function, the first course of amrubicin was initiated as a second-line treatment on April 1, 2019, 17 days after WLI.

### Follow-up

Following the initial course of amrubicin, the patient received two additional courses, with the final administration on June 17, 2019. During this period, hepatic function remained stable, and symptom relief was sustained for approximately 3 months after WLI. His overall performance status improved, allowing continued outpatient management and supportive care. However, on June 27, 2019, 104 days after WLI, CT performed during the evaluation of a lower-limb fracture revealed progression of multiple liver metastases. Because the fracture led to deterioration of his performance status, further chemotherapy was discontinued, and best supportive care was initiated. The patient died on July 27, 2019, 134 days after WLI, in a palliative care unit.

## Conclusions and discussion

WLI has been established as an effective palliative treatment for liver metastases in patients with SCLC. Previous studies, including that by Edyta et al. [6], have demonstrated that a low-dose WLI (mean dose: 17 Gy) provides significant symptomatic relief, achieving a 100% symptom response at 4 weeks and 28% reduction in pain at 3 months in patients with massive liver metastases. However, the therapeutic role of WLI is inherently limited by the liver’s low radiation tolerance, with a maximum dose of approximately 30 Gy [12].

Recent studies have further characterized WLI as a feasible palliative option for diffuse liver metastases from various primary tumors. A retrospective analysis by Ito et al. [13] demonstrated that palliative WLI was well-tolerated and was associated with improvement in hepatic function and symptoms in patients with multiple liver metastases. Kodama et al. [8] also reported a patient with chemotherapy-resistant ED-SCLC who achieved tumor shrinkage and symptomatic improvement after low-dose WLI. In addition, the phase III CCTG HE1 trial showed that radiotherapy to the whole or near-whole liver provided significant pain relief compared with the best supportive care [10]. Together with earlier findings by Ito et al. [7] and Edyta et al. [6], these reports support the continued clinical relevance of WLI as a palliative strategy for selected patients with diffuse liver metastases and severe hepatic dysfunction. In this context, the present case illustrates that WLI may be associated with hepatic recovery sufficient to permit subsequent systemic therapy, although causality cannot be established from a single case report.

In contrast, advancements in radiotherapy techniques, such as stereotactic body radiation therapy (SBRT), have expanded the possibilities for treating liver metastases. SBRT employs high-precision techniques that allow for the delivery of higher radiation doses (70–80 Gy) to limited liver volumes while minimizing toxicity to surrounding tissues [14]. This approach has demonstrated improved local control and survival rates for patients with unresectable liver metastases, particularly in non-SCLC and other solid tumors [12, 15]. While SBRT has well-established eligibility criteria—typically limited to patients with a few discrete hepatic lesions and sufficient residual liver volume—it is not indicated for diffuse liver metastases, such as in this case. Therefore, WLI was selected as a palliative approach to address extensive hepatic involvement. Randomized clinical trials comparing the efficacy and safety of various liver-directed radiotherapy approaches are still needed to provide higher-level evidence [16].

Although supportive care measures, such as intravenous hydration and nutritional management, were concurrently provided, these interventions alone were unlikely to fully explain the rapid biochemical improvement observed. The improvement in liver enzyme levels occurred within 2 weeks after WLI, before the initiation of any systemic chemotherapy. No other interventions specifically targeting hepatic function or liver metastases were administered during this period. However, because this is a single case report, a causal relationship between WLI and hepatic recovery cannot be established. The observed improvement may have been influenced by supportive care, delayed effects of prior chemotherapy, or natural fluctuation of hepatic function in metastatic disease. Nevertheless, the close temporal association between WLI and hepatic recovery, together with the absence of other liver-directed interventions during this period, suggests that WLI may have contributed to the clinical improvement. In addition, formal quantitative symptom assessment using a validated scale was not performed; therefore, the improvement in abdominal distension and appetite was based on the patient’s subjective report.

Other potential causes of hepatic dysfunction were also considered but deemed less likely. Ischemic hepatitis was unlikely, because there was no episode of shock or sustained hypotension. Chemotherapy-induced liver injury was also considered less likely because the hepatic dysfunction worsened approximately 9 weeks after completion of carboplatin plus etoposide and coincided with radiological progression of diffuse liver metastases. Paraneoplastic hepatic dysfunction was considered possible but unlikely, given the marked hepatomegaly and extensive metastatic liver involvement on CT. Thus, diffuse hepatic infiltration by metastatic disease was considered the most plausible cause of hepatic dysfunction.

In this case, we treated a patient with advanced SCLC complicated by diffuse liver metastases and severe hepatic dysfunction using WLI at a dose of 8 Gy in a single fraction for symptom relief. We selected WLI, because the CT images showed significant hepatomegaly, likely caused by micro-metastases extending throughout the liver. Moreover, we selected a dose of 8 Gy, since previous reports demonstrated that even patients with severe hepatic dysfunction (Child–Pugh C) could safely undergo WLI at a dose of 8 Gy, achieving symptom relief and the stabilization of liver function without serious toxicity [13].

Furthermore, in this case, WLI was followed by sustained hepatic stabilization, during which the patient received a total of three courses of amrubicin chemotherapy. Although disease progression was observed 3 months later, this temporary hepatic improvement allowed the continuation of systemic therapy and maintenance of quality of life before clinical decline. Such a course emphasizes the role of WLI as a short-term palliation and supportive measure that can extend the therapeutic window in patients with severe hepatic dysfunction.

This case underscores the unique value of WLI in managing patients with ED-SCLC complicated by severe hepatic dysfunction. Further research is warranted to refine radiation protocols, establish clear patient selection criteria, and optimize the balance between therapeutic benefits and safety.

In conclusion, we presented a case of a patient with ED-SCLC with liver metastases and severe hepatic dysfunction, in whom subsequent chemotherapy was initially considered unfeasible. WLI, administered for symptom palliation, was followed by improvement in hepatic function and subjective symptom relief. This improvement coincided with sufficient hepatic recovery to permit subsequent chemotherapy. SCLC is generally highly sensitive to chemotherapy; however, it progresses rapidly, making systemic chemotherapy the cornerstone of treatment. In selected cases, where hepatic dysfunction caused by liver metastases precludes chemotherapy, WLI may provide symptomatic relief and may be associated with hepatic recovery sufficient to permit subsequent systemic treatment. This case highlights the potential role of WLI as a palliative option for patients with ED-SCLC and severe hepatic dysfunction due to liver metastases. Further studies are warranted to validate its role in improving clinical outcomes in such challenging scenarios.

## Data Availability

All data generated or analyzed during this study are included in this published article. Further inquiries can be directed to the corresponding author.

## Abbreviations

SCLC: Small cell lung cancer
WLI: Whole-liver irradiation
ED-SCLC: Extensive-disease small cell lung cancer
CTCAE: Common Terminology Criteria for Adverse Events
CT: Computed tomography
SBRT: Stereotactic body radiation therapy
